# Multiple Brain Tumor Classification with Dense CNN Architecture Using Brain MRI Images

**DOI:** 10.3390/life13020349

**Published:** 2023-01-28

**Authors:** Osman Özkaraca, Okan İhsan Bağrıaçık, Hüseyin Gürüler, Faheem Khan, Jamil Hussain, Jawad Khan, Umm e Laila

**Affiliations:** 1 Department of Information Systems Engineering, Mugla Sitki Kocman University, Mugla 48000, Turkey; 2 Department of Artificial Intelligence, Mugla Sitki Kocman University, Mugla 48000, Turkey; 3 Department of Computer Engineering, Gachon University, Seongnam-si 13120, Republic of Korea; 4 Daeyang AI Center, Department of Data Science, College of Software & Convergence Technology, Sejong University, Seoul 05006, Republic of Korea; 5 Department of Robotics, Hanyang University, Ansan 15588, Republic of Korea; 6 Department of Computer Engineering, Sir Syed University of Engineering & Technology, Karachi 75300, Pakistan

**Keywords:** healthcare, deep learning, CNN, brain tumor MRI images, image processing

## Abstract

Brain MR images are the most suitable method for detecting chronic nerve diseases such as brain tumors, strokes, dementia, and multiple sclerosis. They are also used as the most sensitive method in evaluating diseases of the pituitary gland, brain vessels, eye, and inner ear organs. Many medical image analysis methods based on deep learning techniques have been proposed for health monitoring and diagnosis from brain MRI images. CNNs (Convolutional Neural Networks) are a sub-branch of deep learning and are often used to analyze visual information. Common uses include image and video recognition, suggestive systems, image classification, medical image analysis, and natural language processing. In this study, a new modular deep learning model was created to retain the existing advantages of known transfer learning methods (DenseNet, VGG16, and basic CNN architectures) in the classification process of MR images and eliminate their disadvantages. Open-source brain tumor images taken from the Kaggle database were used. For the training of the model, two types of splitting were utilized. First, 80% of the MRI image dataset was used in the training phase and 20% in the testing phase. Secondly, 10-fold cross-validation was used. When the proposed deep learning model and other known transfer learning methods were tested on the same MRI dataset, an improvement in classification performance was obtained, but an increase in processing time was observed.

## 1. Introduction

The brain, an organ that searches for meaning and self-inquiry, serves as the center of the entire nervous system to control the body’s other organs. Therefore, any abnormality in the brain can endanger human health. Among such abnormalities, brain tumors, hydrocephalus, and cerebral hemorrhage are the most severe. Brain tumors are divided into primary (primary) and secondary (secondary). Primary brain tumors consisting of the brain’s own cells can be benign (benign) or malignant (malignant). Secondary brain tumors occur when cancerous cells that appear in another part of the body spread to the brain. Primary tumors are located in brain tissue, while secondary tumors expand into brain tissue from other parts of the human body through the bloodstream [1].

According to the World Health Organization (WHO), brain tumors can be divided into four grades. Grade 1 and grade 2 tumors describe lower-grade tumors (e.g., meningiomas), while grade 3 and grade 4 tumors consist of more severe ones (e.g., glioma). In clinical practice, the incidence rates of meningioma, pituitary, and glioma tumors are approximately 15%, 15%, and 45%, respectively [2].

A series of physical and neurological examinations are performed to diagnose brain tumors. Diagnosis is made with MR (Magnetic Resonance) and CT (Computerized Tomography), and biopsy and pathological evaluation are performed to confirm the diagnosis. Among all these imaging modalities, MR is considered the most preferred because it is the only non-invasive and non-ionizing modality [3]. As a result, the type and stage of the cancer are learned precisely, and a treatment plan is prepared. Manual review of medical images in this process is time-consuming, hectic, and even error-prone due to patient flow [4]. To solve this problem in the performed study, it is proposed to develop an automated computer-aided diagnostic (CAD) system to ease the workload of classification and diagnosis of brain MRI and act as a tool to assist radiologists and doctors.

The application was carried out using the Python programming language. Based on free and open-source code logic, Python’s standard library, development tools, and many other libraries can be used free of charge as open-source code without needing a license. Another reason the Python language is preferred is that deep learning libraries used in diagnosing brain tumors exist in Python in the background. The study’s preferred deep learning method for image processing is a subset of machine learning where artificial neural networks and algorithms inspired by the human brain learn from data. When we look at the literature, there are many studies on diagnosing and classifying brain tumors. Different data are collected from different data bases i.e through cloud and big data [5,6,7,8] abnd these data center can be accessed through wired and wirelessly. However, the feature vector must be extracted first to establish a model definition or machine learning system in classical machine learning algorithms. Experts in the field are needed to extract this feature vector. These processes take a large amount of time and keep the expert busy.

For this reason, these techniques cannot process raw data without preprocessing and expert assistance. On the other hand, deep learning has made significant progress by eliminating this problem that those working in machine learning have been dealing with for many years. This was achievable because, unlike traditional machine learning and image processing techniques, deep networks perform the learning process on raw data.

As a result, the literature and experimental research concluded that the models created using transfer learning methods could not achieve the expected success in health image data classification, and there were some areas open to improvement. For this reason, the applied models were examined, and their strengths and weaknesses were reported. Then, a new model structure was created on the CNN’s architectural structure in which the strong points in the models were kept and the weak points were removed. This model architecture uses a convolutional network structure with both dense layers. The proposed new deep learning model improves classification performance and other known transfer learning methods, but an increase in processing time was observed.This study aims to develop a binary classification method for brain tumors (meningiomas and gliomas) and multiclass classification using SoftMax and KNN, non-deep methods with handcrafted features, as well as CNN deep learning methods with transfer learning. The main contributions of the article include:

- In the study, a modified CNN-based system was proposed to increase the accuracy in the classification of brain tumors over previously labeled data.
- Comparison of the proposed model with three different models (simple CNN, VGG16, and ResNet) realized using these labeled data.
- After the models were run, reports were made for each model, and their matrices were created. In this report, the shortcomings of each model were determined.
- In order to eliminate the deficiencies found as a result of the examinations on the models, another CNN model was created, and the errors in the previously examined systems were tried to be eliminated.
- The accuracy rate obtained from studies conducted with labeled data from the Internet has been further increased.
- Medical professionals can use a clinical decision support system to detect brain tumors, which has been developed over the model with increased accuracy.
- Data found on the Internet, previously labeled and made suitable for classification, were used.

## 2. Materials and Methods

The human brain is one of the most complicated organs in our body, with new features being discovered every day. For this reason, many studies on this organ exist in the literature. These can be classified as medical and engineering studies of technology on brain data. For this reason, screening studies related to brain tumors with a wide literature perspectives were limited to the following topics. The following headings were scanned:

- Machine and deep learning algorithms;
- Morphological-based segmentation methods;
- CNN-based classification.

In the study carried out by Gwak et al., a model using deep feature and machine learning classifiers from ensemble learning models was proposed. The study extracted deep features from brain MR images using the transfer learning method in a deep convolutional neural network. Various machine learning classifiers then evaluated the extracted deep features. The top three deep features that perform well in the machine learning classifier are selected and combined into a deep feature collection. Experimental results emphasize that the performance of the ensemble obtained from deep features can help to significantly improve the success of the model [2].

In the study by Asaf Raza et al., a hybrid deep learning model named DeepTumorNet was developed for three types of brain tumors (CT) (glioma, meningioma, and pituitary tumor classification) over a basic convolutional neural network (CNN) architecture. On the GoogLeNet architecture of the CNN model, the last 5 layers were removed, and 15 new layers were added. A Leaky ReLU activation function has been added to the feature map to increase the significance of the model. The proposed model was tested on a public research dataset for evaluation purposes and achieved 99.67% accuracy, 99.6% precision, 100% recall, and 99.66% F1 score [9].

Traditional approaches to image processing are performed by the process of extracting features from the lower layers. However, this algorithm is not particularly suitable for medical images. In their study, researchers named Lakshmi and Rao aimed to detect brain tumors early using the deep learning approach and hyperparameters by using the Inception-v3 convolutional neural network model. In the study, it is seen that the accuracy of the Inception-v3 algorithm was recorded as 99.34% in the training data and 89% in the validation data [10].

In the study by Chenjie Ge et al., a model using a graph-based semi-supervised learning method was proposed to benefit more from unlabeled data. The proposed model was tested on two glioma datasets, the TCGA dataset for IDH mutation prediction and the MICCAI dataset for glioma grading. It has been reported that an 86.53% test accuracy was obtained in the TCGA dataset and 90.70% in the MICCAI dataset [11].

As can be seen from the literature, many studies have been carried out on brain tumors in recent years. It can be seen that there are many medical imaging techniques for the diagnosis of these tumors [12]. It has been concluded that most of the previous studies were on the segmentation of areas of brain tumors, but recent studies have focused on classifying these areas into different types of brain tumors. Based on this, the study was carried out to increase the success rate of the binary classification of brain tumors.

During the literature research phase of the study, many recent studies were examined. The most striking of these studies are presented in Table 1. These studies draw attention because the issues to be considered in the model to be created are included. The table below discusses the models used in the studies, the success rates, and the results obtained from the study.

## 3. Methodology

### 3.1. Overview

Convolutional neural networks are a sub-branch of deep learning and are often used to analyze visual information. CNN or ConvNet is short for convolutional neural network. Convolutional neural networks consist of many layers that can be trained. These are the input, convolution, jointing, and full link layers. The convolution layer and the jointing layer can be fine-tuned with hyperparameters. Different CNN architectures combined with different transfer learning techniques have achieved great success thanks to their improved performance in image classification. In this way, they have surpassed traditional machine-learning models in the last few years. CNN algorithms can automatically recognize much more important visual patterns with less preprocessing on the raw pixels of images. Serving as a test environment, ImageNet has played an important role in advancing deep image recognition architectures. CNN algorithms started to be used rapidly with the ImageNet Large Scale Visual Recognition Challenge (ILSVRC) competition, which was first held in 2012 [22].

Another CNN architecture, VGG16Net, was ranked second in ILSCRC for accuracy in 2014 [18]. The largest created VGG16 architecture consists of 16 layers, 3 of which are fully connected and contain an average of 144 million parameters. The layers contain five pooling layers 2 × 2 in size in each convolution layer. There is also a softmax linear layer at the output. The ReLU activation function is applied in all fully connected layers, and at the same time, the Dropout Layer is used in fully connected layers. Compared to popular methods, it is a CNN model that is considered to have a high computational load due to its large parameters.

**Figure 1:**
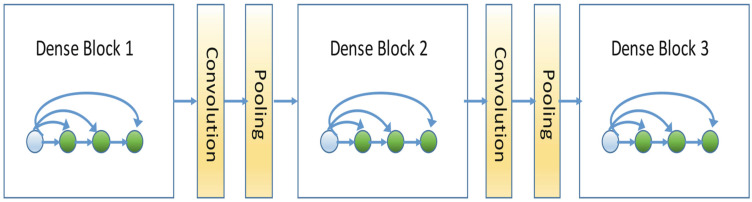
Three-Block DenseNet Architecture.

DenseNet is a recently proposed custom convolutional neural network model where the current layer connects with all previous layers [23]. The structure has some advantages over existing structures, such as mitigating the disappearing gradient problem, enhancing feature propagation, promoting feature reuse, and reducing the number of parameters. A deep DenseNet is defined as a series of DenseNets (called dense blocks) sequentially connected by additional convolution and pooling operations between consecutive dense blocks. We can create a deep neural network flexible enough to represent complex transformations with such a structure. An example of deep DenseNet is shown in Figure 1.

### 3.2. Methods and Materials 

**Figure 2:**
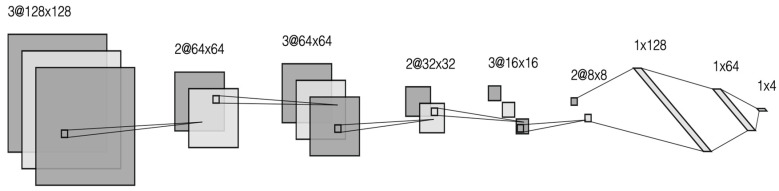
Modified CNN Architecture.

Some changes were made in the modified CNN model, taking into account the shortcomings of other models. As a result of the examination of the VGG16 Net architectural structure, it was observed that the VGG16 Net convolutional layers positively affected the success rate in areas where health data are not used. For this reason, it was decided to use convolutional layers in the model created. Afterward, the DenseNet architecture was examined. Thanks to the dense layers of this architectural structure, it was determined that a more detailed search was carried out in the MRI images. For this reason, convolutional networks were built with a more intense working principle in the model created. Finally, a decrease in the success rate was detected due to the low number of layers in the analysis of the CNN’s simple architectural structure. For this reason, the number of layers was increased. In this way, searches of the image were made more detailed. The adjustments made in the created model and the increase in the number of layers affected the classification rates and increased the success rate. The Figure 2 explain the components of modified CNN networks.

After the model was created, two different paths were chosen for training and testing. First, the data set was trained in a classical way and separated as a test. Then, the training data were used during deep learning. Afterward, the test data and the success rates of the model were examined. The K-fold cross-validation method was chosen as a second method, and K was determined as 3. The reason for choosing this method was to verify the model’s reliability by creating independent data sets and to prove that the model will show the same success in all kinds of data sets. 

During the material selection stage, research was carried out from many sources. As a result of the research, more than 7000 images shared as open source on Kaggle and intended for use in brain tumor image processing studies were obtained. These images consist of 4 different classes in total, classified as three different types of brain tumors and images of healthy individuals. Later, these data were divided to be used in the training and testing phases. The purpose of this was to not use the data used in the training phase in the testing phase. Such a distinction has been made because reclassifying the data the model has seen before during machine learning would affect the accuracy rate. Afterward, studies using the same data set on Kaggle were examined. The reason for these studies was to identify the deficiencies in other studies and to eliminate these deficiencies in our study. Three popular studies were examined. Two of them are models that have been trained by machines before and are expected to have a high success rate. Finally, a simple CNN architecture was examined. This review aims to measure the success of the CNN architecture, which is generally used in image processing in the health field, and then to determine how contributions should be made to the modified CNN architecture.

#### 3.2.1. K-Fold Cross Validation

An extra k-fold cross-validation method was added to test the model’s accuracy and examine the results. This method was added to retrain and test the model with independent data sets other than manually separated data sets. 

Cross-validation is a model validation technique that tests how a statistical analysis will yield an independent data set. Its primary use is to predict with what accuracy a prediction system will work in practice. In a prediction problem, the model is usually trained on a set of “known data” (“training set”) and tested against a set of “unknown data” (“validation set” or “test set”). This test aims to measure the trained model’s generalization ability to new data and to detect overfitting or selection bias problems. Simple approach: Set aside 75% for training and 25% for testing. However, while the data are fragmented, some biases and errors may occur in the training and testing of the model, depending on the distribution of the data. Here, k-fold cross-validation divides the data into equal parts according to a determined k number, ensuring that each part is used for both training and testing, thus minimizing deviations and errors caused by dispersion and fragmentation. The K value is usually chosen as 3 or 5. This value can be selected as 10 or 15, but this will cause a costly calculation and time loss.

#### 3.2.2. Dataset

**Figure 3:**
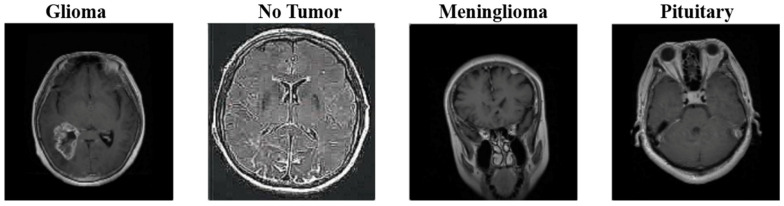
Classes of the Dataset.

An open-source brain tumor dataset was used to analyze and evaluate our model, which was developed using different CNN architectures, as shown in Figure 3. This dataset was obtained by combining three datasets (figshare, SARTAJ dataset, Br35H) [24]. There are four classes in total in the data set. These are: brain MRI images from glioma, meningioma, oituitary, and healthy individuals. There are 1623 images for glioma, 1627 images for meningioma, 1769 images for pituitary, and 2002 images for healthy individuals. A total of 7021 MRI images were used. The dataset is open-sourced in the Kaggle application. Each file is a 512 × 512 JPEG file with a label indicating the type of brain tumor. This data set was used as input data for each model.

For classifying and defining the brain tumor using the MRI images of the brain tumor dataset, three different models were first processed; then, the model created with these models was compared. Examined models included: standard CNN architecture, VGG16Net architecture, and DenseNet architecture. These models were compared with the later modified model.

## 4. Results and Discussion and Evaluation of Parameters

The effectiveness of the proposed brain tumor classification and detection system is evaluated by calculating the four main outcome-based evaluation metrics used to test the classifier: true positives (TP), false positives (FP), true negatives (TN), and false negatives (FN). The performance of the proposed system is evaluated using the following parameters, as can be seen in Table 2.

Accuracy determines the ability to accurately distinguish brain tumor types. To estimate the accuracy of a test, we calculate the ratio of true positive and true negative for all evaluated cases calculated by the following relationships:

$$Accuracy=\frac{TP+FN}{TP+TN+FP+FN}$$

On the other hand, recall is a metric that shows how many of the operations we need to estimate as positive are positive.

$$Recall=\frac{TP}{TP+FN}$$

Precision, on the other hand, shows how many of the values we estimated as positive are actually positive:

$$Precision=\frac{TP}{TP+FP}$$

The F1 Score value shows us the harmonic mean of Precision and Recall values. The reason why it is a harmonic mean instead of a simple mean is that we should not ignore the extreme cases. If it were a simple average calculation, a model with a Precision value of 1 and a Recall value of 0 would have an F1 Score of 0.5, which would mislead us:

$$F_{1}=\frac{2*Precision*Recall}{Precision+Recall}$$

### 4.1. Training Parameters

The training options and hyperparameter networks assigned for training are listed in Table 3. Here, the same training options are used to compare the performance of different architectures. Networks are trained at a learning rate of 1010 for 100 epochs. The data set was divided into two training and testing. A total of 5650 data points were used in the training phase, and 1371 data points were used in the testing phase. The model was trained for 100 epochs. The horizontal axis refers to the number of epochs, and the vertical axis refers to the error rate.

### 4.2. Training Phase

As a result of the research carried out on Kaggle, three different networks were selected to be examined. These networks are Basic CNN, VGG16Net, and DenseNet. Then, the data percentage to be used in the training phase was determined on the chosen data set: 80% of the data is reserved for use during the training phase and 20% for the testing phase. During the training phase, the accuracy rates of architectural structures, validation accuracy rates, and changes in loss functions were examined and tabulated at each step. These changes were examined separately for each model. It was learned that the same errors occurred in certain classes during the classification in the previously trained models. In the simple CNN network, classification errors arose due to deficiencies in the layers. These errors were reduced to minimum levels in the modified CNN network, and high accuracy rates were obtained during the training phase.

#### 4.2.1. Basic CNN Architecture

During the literature research phase of the study, the models in other studies were examined. As a result of this research, it was concluded that the popularities of the classical CNN model, VGG16Net, and DenseNet models in the studies were high, so it was decided to examine studies of these models. In the classical CNN architecture, firstly, the structure of the layers was examined. There is only one convolutional layer and a pooling layer in this structure. Then, classification is performed by passing information to the classical neural network. In the proposed approach, convolutional and pooling layers have been developed and inserted into the classical neural network after multiple preprocessing steps.

As a result of the research carried out on Kaggle, it was decided that the first structure to be examined would be a simple CNN architecture. The purpose of choosing this architectural structure is to observe how successful the CNN architecture will be without changing the layers. As a result of these observations, it was decided how changes should be made in the layers. The system was operated with a total of 10 epochs, and the resulting values were examined. These values are shown in Table 4.

**Figure 4:**
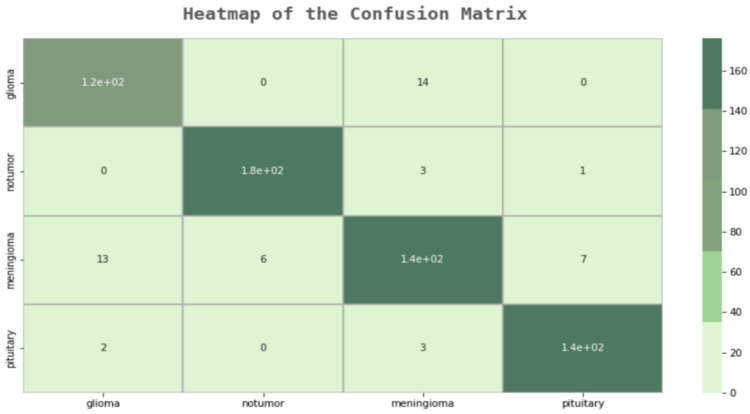
Basic CNN Confusion Matrix.

As can be seen from the graphics, the accuracy of the system is up to 92%. However, to test the reliability of the system, an application was made using the test data set. The reason for this is that even if the success rates of the created models are high during the training phase, their inadequacy in the testing phase has been observed before. As a result of this application, and as can be seen in Figure 4, an error matrix was created, and Precision, Sensitivity, and F1 Scores were examined over this error matrix.

As can be seen from the error matrix in Figure 4, the simple CNN model works well for two classes (Healthy Individual and Pituitary). However, the success rate in other classes was less than expected. Even if the success rate in other classes is sufficient as a result of a normal examination, a success rate of 89–90% is not sufficient in a study on cancer in the field of health. For this reason, this model is not a model that can achieve the necessary success in real life. The weak points of this model were examined, and it was determined which parts were missing. Even though the success rate in MRI images of healthy individuals and patients with the pituitary disease was at the expected rate, the success rates in MRI images in the glioma and meninglioma tumor classes were below the expected levels.

In order to examine the reliability of the model in detail, the values of each tumor class at the test stage were examined separately. In this way, it has become easier to detect the model’s weak points. As we have previously examined in the error matrix, although the success rate in healthy individuals and patients with a pituitary brain tumor was 97–98%, the success rate in meninglioma and glioma tumor types remained below 90%, as shown in Table 5. This ratio is not sufficient for application in the field of health. The reason for this low success rate is the equation we use to calculate the F1 score. While calculating the F1 score, the value that affects the ratio the most is the “False Positive” value. This value occurs when people’s test results are negative, but they have a disease, and it is the most important value in medical applications. This is the weakest point of this system since this value is too high in the case of meninglioma.

#### 4.2.2. VGG16Net Architecture

The VGG16Net architectural structure uses convolutional layers in groups of two or three, and it is different from CNN model structures. At the same time, the VGG16Net architecture functions as a previously trained model. The reason for making a comparison between VGG16Net and the proposed model is that the complexity in the former’s network structure is higher than the proposed model. We also want to see how much a pre-trained model will affect the success rate.

As the second model, it was decided to examine the VGG16Net architectural structure. A new model was created using the VGG16 transfer learning method. The purpose of this model is to increase its reliability by applying training with large datasets. This model was run at the same epoch number with the simple CNN architecture, and its values during the training phase are given in Table 6.

As seen in Table 6, the accuracy rate of the model regularly increases throughout the training phase. However, during the validation stage, that is, the pre-test phase, the accuracy values could not meet the expected values and did not show a regular increase. The validation accuracy decreased again in some epochs. Afterward, the trained model was tested, and an error matrix was created. A table was created to examine this matrix in detail and to see the “Sensitivity,” “Precision,” and “F1 Score” values. As seen on the confusion matrix, the VGG 16 Model worked with a lower success rate than the simple CNN model, and the success rate decreased in the same classes. The model that made many mistakes in the glioma brain tumor class was determined as a model that is unlikely to be used in the health field as a success rate.

**Figure 5:**
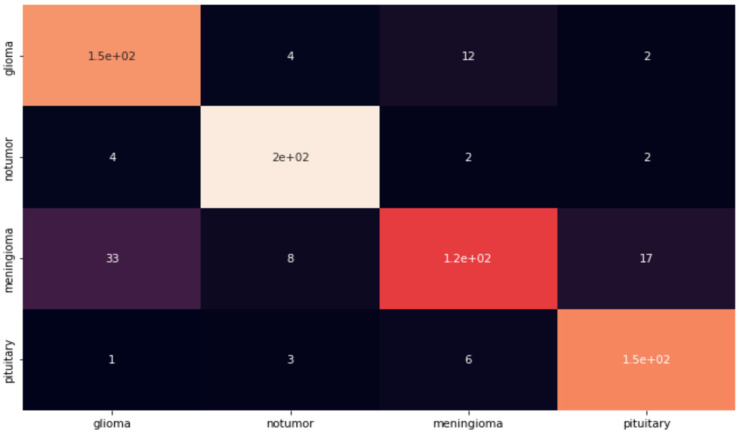
VGG16 Architectural Structure Confusion Matrix.

Later, when we examined the performance of Table 7 according to their classes, it made too many mistakes and examined the deficiencies in meninglioma and glioma tumor types similar to the previous model. It did not achieve the expected success in distinguishing tumor classes at the classification stage, as shown in Figure 5.

#### 4.2.3. DenseNet Architectural Structure

The reason for examining the DenseNet architecture is that in DenseNet, each layer receives inputs from the previous layers and transfers all feature maps to the next layers. The aim here is to compare the CNN architectural structure and the DenseNet architectural structure, which transfers collective information. This way, the operation of feature maps will be compared, and the differences between the models will be examined.

Finally, a different transfer learning model was examined based on the VGG16 transfer learning model. The reason for choosing the DenseNet architecture is that it can better learn the details in the inputs and outputs by using dense layers. The DenseNet model is also a model that has been previously trained with different data sets. It was run on the same data set, with the same number of epochs as the other models. The values at the training stage are given in Table 8.

Like the VGG16 model, the DenseNet model showed a regular increase in accuracy during the training phase. However, also like the VGG16 model, there is a tide in the accuracy rates in the pre-test phase, causing a loss of confidence. After some epochs, there were decreases. In order to examine the success rates in the test phase, a table showing the “Sensitivity”, “Precision,” and “F1 Score” values was created.

As can be seen in Table 9, unlike the VGG16 architecture, DenseNet’s architecture failed to detect meninglioma and glioma tumors, although the Sensitivity ratio was high in healthy individuals and pituitary tumor patients. Likewise, the apparent insufficiency in F1 scores is due to the high number of “False Positives.”

#### 4.2.4. Modified CNN Architecture

As a result of the examination of the three models, deficiencies in each model were determined. Afterward, a CNN model was created in order to eliminate these deficiencies. In this model, unlike the simple CNN model, a more advanced network is created, and images are analyzed in more detail in this network. The values of the model operated in the form of 20 epochs during the training phase are given in Table 10.

As can be seen in Table 10, a 99% success rate was achieved in the training phase, unlike other models. In the pre-test phase, this rate increased to 95%. In order to test this model and examine in which parts it makes mistakes, an error matrix, followed by a “Sensitivity”, “Precision”, and “F1 Score” table, was created.

**Figure 6:**
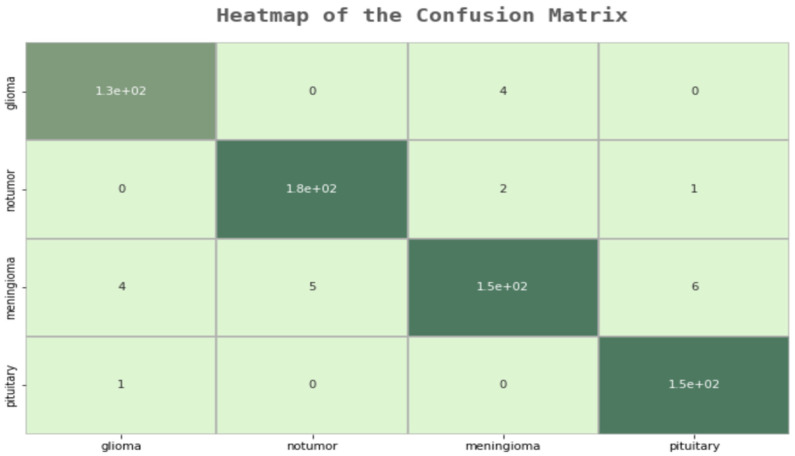
Modified Model Confusion Matrix.

As a result of the examinations on the error matrix, all MR images were correctly classified, except for 23 images in total. A “False Positive”, which is the most important for us, was only made in three images. In the developed model, many of the deficiencies of the other models were eliminated, and the F1 score was examined to check the model’s reliability, as shown in Figure 6. In Table 11, additional evaluation metrics are provided for each class. These metrics convey useful information regarding the modified model’ predictive power for each class.

In order to prove this increase and to ensure the reliability of the model, a K-fold cross-validation method was also added, and the results of this method were examined. As a result of the cross-validation process, it has been proven that the model’s work with independent data sets does not reduce its success rate, and it works better than other models. 

In the K-fold cross-validation, K was determined as 10. In the first stage, K was determined as five, and the results were examined. After the K-fold application, an increase in the success rate was observed. The reason why five-fold cross-validation was chosen first is to determine how it would affect the operation of the system. Later, due to the increase in the success rate, K was determined as ten and run again. The reason for this was to observe whether there would be an increase in the success rate as a result of 10-fold cross-validation. However, success rates continued to rise between 97 and 95. It was decided to use Google Colab during the operations. This is because the model that is running on Jupyter has been shown to have a very long processing time. The fast transaction service provided via Google Colab was utilized. The comparative results for the brain tumor databases are depicted in Table 12.

The developed CNN architectural structure showed a higher success than other architectural structures. The classical CNN architectural structure could not reach the desired success rate due to the low number of layers. In the examination of the model, it was observed that there were some deficiencies in feature extraction due to the number of layers, and for this reason, the success rate decreased in some classes.

In the VGG16Net architectural structure, it has been observed how to measure the success rate of pre-trained models and how increasing the number of convolutional layers will affect the success rate. It was concluded that the previously trained models had a low overshoot rate on health data. For this reason, the margin of error is high in the classification stage of such architectural structures. 

Finally, the reason for examining the DenseNet architectural structure is to observe how the use of denser layers in the transfer learning method and the collective knowledge transfer will yield results. Although dense layers are more effective than the VGG16Net architectural structure, the error rate was high in some classes due to the transfer learning method. 

The conclusion that is understood as a result of these examinations is that the layers should be used intensively and that the training phase should be carried out without using transfer learning methods. For this reason, an intensive CNN model was created in the proposed method, in which 80% of the data was used in the training phase and the model was self-trained without using transfer learning methods. 

Table 13 was created to compare and examine the average values of precision, sensitivity, and F1 Scores of all models

Table 14 gives average evaluation metrics (F1 Score, Precision, and Recall) for a comparison of the results having the same dataset as the state-of-the-art studies.

When the table is examined, it can be observed that our research is in close proximity with the studies that reach high accuracy among modern studies. There is also some consistency between all three scales. Detailed interventions that can be carried out in the data preparation and cleaning stages can raise the values a little higher.

## 5. Conclusions

In our study, researchers determined weak points by examining many image processing methods. These studies were conducted to examine the missing parts of previous studies and eliminate these deficiencies. First, the simple structure of the CNN architecture was examined, and some tests were carried out using our study’s dataset. As a result of these tests, it has been determined that there are some deficiencies in classification due to the small number of layers. Afterward, the VGG16Net and DenseNet architectural structures were examined. These models were examined to determine how the transfer learning method would affect their success rates and to observe how the use of dense layers will contribute to them. It has been observed that the transfer learning method does not affect the health field’s success rate, even if the success rate is high in other areas. For this reason, it was decided not to use the transfer learning method in the model created in this study. Afterward, DenseNet analysis was carried out. Although it affected the success rate positively in dense layers, the success rate did not increase to the expected level due to transfer learning. For this reason, it was decided that a structure with dense layers should be created, and the training phase should be completed in person. A CNN architecture was used in the created model, and the density of the layers in the preprocessing stage was kept at a high rate. In this way, the rate of obtaining information from the data has increased. Since it was decided not to use the transfer learning method, it was concluded that more data were needed. For this reason, as a result of the research, a dataset published as open source on Kaggle was found. In this dataset, there are four classes, namely, Glioma, Meninglioma, Pituitary, and No Tumor, and these classes contain more than 7000 images in total. The dataset is divided into 80% training and 20% testing phases. The expected success rate was achieved due to the large amount of data in the training phase and the fact that the proposed model was created with dense layers. This way, a successful training phase was achieved thanks to the dense layers without using transfer learning methods, and a success rate of 94–97% was achieved. In future studies, it is aimed to improve the weak points of our model. The most important weakness of the proposed model is the long processing time. The reason for the late response is that the model was created using both dense and convolutional networks. Afterward, we would like to add segmentation areas with tumors. This way, after the tumor classification is complete, it will be possible to calculate in which part of the brain the tumor is located and its size. If these goals are completed, a system will be created that will help people working in the field of health to lighten their burdens.

## Figures and Tables

**Table 1:** Comparative Analysis.

Ref.	Year	Methodology	Dataset	Result	Drawback
[13]	2020	PNN Classification CNN	KaggleTCIA	%90 Accuracy	Lack of comparative analysis
[14]	2019	Inception Pre-Trained CNN	BRATS 13,14,17,18	%99.12 Accuracy	Complex Approach
[15]	2019	CNN with a Modified Softmax Loss Function	BRATS, ISLES, FLAIR, DWI	%98.9 Accuracy	It can be developed and can be tested with large datasets.
[16]	2018	ELM-LRF	MNI Brain Website	%97 Classification Success	More efficient than related work because training time is shorter than others
[17]	2029	CNN	TCIA, Kaggle	%90 Accuracy	Long Process Time
[18]	2022	CNN	Harvard Medical School website	%98.5 Accuracy	Computational time, system complexity, and memory space requirements are too much
[19]	2022	SVM	BRATS 2017	%92.3 Accuracy	SVM cannot handle a larger data set.
[20]	2022	Evolutionary CNN	BRATS 2015 data set and brain image datasets from Harvard Medical School,	%97.4 Accuracy	Complex System
[21]	2021	HOG + LBP + deep features	BRATS2015	%96 DSC	The problem in thresholding point

**Table 2:** Confusion matrix definition.

Class	Statement	Formula
True Positive	Images that belong to a patient who is sick and correctly known by the model	TP
True Negative	Images that belong to a patient who is healthy and correctly known by the model	TN
False Positive	Images that belong to a patient who is healthy but diagnosed as sick by the model	FP
False Negative	Images that belong to a patient who is sick but diagnosed as healthy by the model	FN

**Table 3:** Hyperparameters.

Parameters	Value
Momentum	0.9
Initial learning rate	10^−4^
Maximum number of epochs	100
Validation Split	0.1

**Table 4:** Simple CNN Architecture Training Stage Accuracy Values.

Number of Epochs	Loss Function	Accuracy Rate	Validation Loss Function	Validation Accuracy Rate
1	1.2098	0.6435	1.8210	0.6485
2	0.5977	0.7582	0.5531	0.7680
3	0.4806	0.7959	0.6529	0.7680
4	0.4826	0.8154	0.4741	0.8629
5	0.3804	0.8502	0.5892	0.7856
6	0.3072	0.8685	0.4932	0.8084
7	0.2291	0.8969	0.3390	0.9086
8	0.2102	0.9099	0.3310	0.9174
9	0.1953	0.9166	0.3505	0.9033
10	0.1732	0.9232	0.3290	0.9192

**Table 5:** Performance of the CNN model by class.

Brain Tumor Classes	Precision	Recall	F1 Score
0	0.89	0.90	0.89
1	0.97	0.98	0.97
2	0.88	0.85	0. 86
3	0.95	097	0.96

**Table 6:** VGG16 Architecture Training Phase Accuracy Values.

Number of Epochs	Loss Function	Accuracy	Validation Loss Function	Validation Accuracy Rate
1	8.3011	0.5701	4.1341	0.7255
2	2.8375	0.7731	2.8020	0.7824
3	1.9171	0.8286	2.1238	0.8023
4	1.4598	0.8527	1.7471	0.8222
5	1.1312	0.8698	1.6981	0.8293
6	0.9522	0.8785	1.6033	0.8222
7	0.7834	0.8905	1.2539	0.8478
8	0.6701	0.9013	1.1761	0.8592
9	0.5615	0.9051	0.9738	0.8720
10	0.4940	0.9103	0.9641	0.8663

**Table 7:** VGG16 Model Performance Table by Classes.

Brain Tumor Classes	Precision	Recall	F1 Score
0	0.80	0.89	0.84
1	0.93	0.96	0.94
2	0.85	0.67	0.75
3	0.83	0.94	0.90

**Table 8:** DenseNet Architectural Structure Education Phase Values.

Number of Epochs	Loss Function	Accuracy Rate	Validation Loss Function	Validation Accuracy Rate
1	0.5760	0.7787	0.3221	0.8766
2	0.4070	0.8427	0.3887	0.8574
3	0.3460	0.8634	0.4153	0.8574
4	0.3095	0.8855	0.3364	0.8775
5	0.3030	0.8879	0.3355	0.8836
6	0.2894	0.8828	0.3757	0.8688
7	0.2783	0.8993	0.3116	0.8889
8	0.2747	0.8958	0.3682	0.8329
9	0.2507	0.9068	0.2559	0.9064
10	0.2504	0.9030	0.3787	0.8608

**Table 9:** DenseNet Architectural Structure Performance Values by Class.

Brain Tumor Classes	Precision	Recall	F1 Score
0	0.75	0.99	0.85
1	0.91	0.99	0.95
2	0.93	0.83	0.88
3	0.92	0.58	0.71

**Table 10:** Modified Model Training Stage Values.

Number of Epochs	Loss Function	Accuracy Rate	Validation Loss Function	Validation Accuracy Rate
1	0.7699	0.7271	1.1123	0.6538
2	0.4267	0.8429	0.6523	0.7522
3	0.3269	0.8773	0.7634	0.7135
4	0.2142	0.9228	0.3145	0.8981
5	0.1867	0.9310	4.2310	0.6520
6	0.1422	0.9496	0.5562	0.8418
7	0.0750	0.9742	0.2394	0.9262
8	0.0599	0.9797	0.5438	0.8453
9	0.0493	0.9850	0.1974	0.9455
10	0.0472	0.9855	0.1936	0.9438
11	0.0404	0.9877	0.2002	0.9438
12	0.0383	0.9877	0.1982	0.9525
13	0.0317	0.9922	0.2054	0.9438
14	0.0342	0.9887	0.2039	0.9455
15	0.0342	0.9914	0.2040	0.9473
16	0.0336	0.9910	0.2056	0.9438
17	0.0312	0.9910	0.2050	0.9438
18	0.0323	0.9910	0.2045	0.9455
19	0.0390	0.9895	0.2048	0.9455
20	0.0304	0.9914	0.2046	0.9455

**Table 11:** Performance Values by Modified Model Classes.

Brain Tumor Classes	Precision	Recall	F1 Score
0	0.96	0.97	0.97
1	0.97	0.98	0.98
2	0.96	0.91	0.94
3	0.95	0.99	0.97

**Table 12:** Ten-fold cross-validation performance rates (%).

Fold Number N	Training Accuracy	Test Accuracy
1	0.96	0.94
2	0.95	0.93
3	0.96	0.94
4	0.95	0.9256
5	0.97	0.94
6	0.96	0.93
7	0.95	0.94
8	0.97	0.94
9	0.97	0.95
10	0.96	0.94

**Table 13:** Comparison Table of Models.

Model Name	Number of Epochs	Avg. Precision	Avg. Recall	Avg. F1 Score
Basic CNN	10	0.9225	0.9	0.92
VGG16 Net	10	0.8625	0.8625	0.8575
DenseNet	10	0.8775	0.8775	0.8475
Modified CNN	10	0.96	0.96	0.9650

**Table 14:** Comparison Table of Models with other papers.

	Raza et al. [9]	Deepak & Ameer [25]	Alqudah et al. [26]	Saleh et al. [27]	Ghassemi et al. [28]	Our study
F1 Score	99.66	95.34	98.91	99.75	95.09	96.00
Precision	99.60	94.70	98.98	-	95.28	96.00
Recall	100	96.00	98.85	-	94.91	96.50

## Data Availability

Not applicable.
